# Parents’ experiences of seeking health care and encountering allegations of shaken baby syndrome: A qualitative study

**DOI:** 10.1371/journal.pone.0228911

**Published:** 2020-02-20

**Authors:** Ulf Högberg, Gunnel Eriksson, Göran Högberg, Åsa Wahlberg

**Affiliations:** 1 Department of Women’s and Children’s Health, Uppsala University, Uppsala, Sweden; 2 Department of Epidemiology and Global Health, Umeå University, Umeå, Sweden; 3 Formerly of The Regional Psychiatric Clinic of Jämtland—Härjedalen, The Adult and Adolescent Units, Östersund, Sweden; 4 Formerly of the Department of Women’s and Children’s Health, Child and Adolescent Psychiatric Unit, Karolinska Institutet, Stockholm, Sweden; 5 Department of Psychiatry, Visby County Hospital, Visby, Sweden; Institute of Mental Health, SINGAPORE

## Abstract

**Objectives:**

To explore parents’ experiences of seeking health care for their children and instead being accused by healthcare professionals of Shaken Baby Syndrome/Abusive Head Trauma (SBS/AHT), being reported to Social Services, undergoing judiciary processing, and the impact of these events on family (dis)integration.

**Methods:**

Design: A qualitative study based on qualitative content analysis. Participants: Twelve parents in Sweden, mothers and fathers, seeking health care for their infants, encountering allegations of SBS/AHT, losing custody of their infants, and being subjected to a judiciary process, and finally regaining custody of their children. Data collection: In-depth interviews.

**Results:**

An overarching theme ‘Fighting for protection of their child after being trapped by doctors’ and four sub-themes were developed to reflect the parents’ experiences, reactions and interpretations. The first sub-theme, ‘Being accused of injuring the child’, illuminated the shock experienced when seeking care and instead being accused of being a perpetrator. The second, ‘Chaos and powerlessness’, refers to the emotions experienced when losing custody of the child and being caught in the enforcement of legislation by the authorities. The third, ´The unified fight against the doctors’ verdict´, illustrates the parents’ fight for innocence, their worry for the lost child, and their support and resistance. The fourth, ‘The wounded posttraumatic growth’, describes the emotions, grief, panic, anxiety, and challenges in reuniting the family, but also the parents’ reflections on personal growth. Unanimously, they had experienced the authorities’ inability to reconsider, and expressed a deep mistrust of paediatric care.

**Conclusions:**

Being wrongly accused of child abuse and alleged SBS/AHT evoked emotions of intense stress, but parents endured because of a successful fight to regain custody of their child. However, the trauma had a long-term impact on their lives with residual posttraumatic stress symptoms and mistrust towards healthcare services and the authorities. The results provide important inferences for restoring system failures within child protection services.

## Introduction

In the best interests of the child, correct diagnosis of infant abuse comprises the ethical principles of beneficence, non-maleficence and justice. Misclassification and missed cases of abuse, or wrongly accusing parents, can have severe consequences. Existing research has been focussed on barriers among doctors and nurses to detect and report suspected abuse, such as inadequate training, both a lack of and uncertainty within guidelines, a lack of support from authorities [1, 2], disturbances within the doctor-patient relationship [1, 3], mistrusting the benefit to the child and family when abuse is reported [2, 4], and previously established relationships and knowledge about the parents as family doctor when accepting the parents’ explanation for an injury [2]. Sentinel cases experienced earlier in practitioners’ careers [5], and media attention relating to undetected cases of child abuse with fatal consequences, might also enhance a climate of fear, mistrust and blame, leading to a defensive proceduralization and technicalization of child protection [6] where false-positives cases might also occur [7].

The diagnosis of shaken baby syndrome/abusive head trauma (SBS/AHT) has been described as within the “second wave of child protection”, characterized by developments in techno-science [8]. The concept of shaken baby syndrome derived from the description of the battered child syndrome in 1962 [9], that shaking was hypothetised to cause subdural hemorrhage in 1971 [10], the description of the whiplash shaken infant syndrome adding retinal haemorrhage and encephalopathy to the triad criteria in 1974 [11], and further rib fractures and long bone fractures as CMLs (classical metaphyseal lesions) to be caused by shaking and added to the criteria [12]. The Committee on Child Abuse and Neglect of the American Academy of Pediatrics prefer since 2009 to use the term abusive head trauma, instead of shaken baby syndrome [12] valid for the above mentioned criteria for diagnosing infant abuse (9). By employing imaging technology, a doctor formulates the event that has happened, when it happened, and can determine whether someone has performed the act, all based on the medical findings of radiology or eye examination. However, a systematic review by the Swedish Agency for Health and Technology Assessment and Assessment of Social Service (SBU) in 2016 showed there is insufficient scientific evidence to assess the diagnostic accuracy of the triad (subdural haemorrhage retinal haemorrhage and encephalopathy) in identifying traumatic shaking, mainly because of circular reasoning [13]. Although it has been criticized, its scientific conclusions have not been changed [14]. We have further provided evidence that infant morbidities, such as subdural haemorrhage [15] and fractures [16], share risk factors with those diagnosed as non-accidental injury. Error rates in issuing false abuse labels are not reported [17].

Compared to other Western countries, Sweden has a lower incidence of Out-of-home care for infants [18]. Additionally, a very low percentage is associated with Shaken baby syndrome/abusive head trauma criteria (SBS/AHT) compared to other countries [18]. However, in Sweden, this number has increased considerably during this century [19]. A recommendation when a case of SBS/AHT is detected is that foster care should be lifelong [20].

Research is scant on parents’ experiences of when Social Services take their children into custody. One study from Australia reports questions of human rights abuse [21], and one study from Canada reports the experiences of child custody loss as a profoundly traumatic event, producing long-lasting impact on the parents’ lives [22]. To our knowledge, only one study has examined experiences of loss among parents whose children were in child protective services because of false allegations of abuse or neglect. The results describe four themes of loss: the physical separation, their ability to protect their child, their faith in the system, and their confidence in their parenting ability [23]. The US lawyer, Diane Readleaf, has reported on a series of cases where parents are struck, as if by an earthquake, when they have been accused of shaking their babies [17].

In case of an eventual over-diagnosis of infant abuse, there is a need to listen to parents’ experiences of health and social care and of the judicial process related to alleged infant abuse to gain a patient/client perspective about possible inflicted harm in health and social care. To our knowledge, no study so far has addressed both mothers’ and fathers’ experiences of seeking health care for their infants and instead being accused of shaking their babies.

### Objective

The objective was to explore the experiences of parents who sought health care for their children yet were accused by healthcare professionals of SBS/AHT, then were subjected to inquiries by judicial and Social Services, and the impact of these events on family (dis)integration, whereby children were taken into custody and fathers were convicted, but later exonerated.

## Material and methods

### Study design

The study design was qualitative, using qualitative content analysis [24, 25]. This approach was chosen as it is suitable for understanding the lived experiences of a phenomenon. In-depth interviews were chosen as the main data collection method as they are suitable for exploring individuals’ experiences [26].

### Study setting

Sweden has a long history of well-developed child welfare and child protection services, and, compared to other Western countries, a low and declining incidence of child abuse, apart from infant abuse, over the years, as illustrated by longitudinal data collected until 2009 [18]. In 2007, the Child Protection Team was established at Karolinska Sjukhuset. This group developed guidelines for SBS/AHT-screening that have since been disseminated among paediatricians [27]. Additional Child Protections Teams were then established in five Swedish Paediatric University Hospitals [28]. In Sweden, the incidence of diagnosis of infant abuse is strongly associated with the introduction of the criteria for SBS/AHT, and has doubled from the total number in the period 1997–2007 to the total for 2008–2014 with pronounced regional disparities [7]. In 2014, the Swedish Supreme Court ruled that SBS/AHT findings alone cannot be grounds for the conviction of a parent for infant abuse and, since then, several cases of acquittal of previously sentenced fathers who were convicted of SBS/AHT against their infants have been granted by the Court of Appeal. Furthermore, in 2018, The Swedish Supreme Administrative Court ruled that medical findings according to the SBS/AHT-criteria cannot solely be cited as a justification for ordering Out-of-home care.

### Study population and sampling of informants

The research group contacted 20 parents (10 mothers and 10 fathers) through an informal internet peer support and advocacy group (www.rffr.se) with an invitation to be interviewed and to participate in a qualitative study. Eight of those declined and 12 (6 mothers and 6 fathers) agreed. Events relating to the parents’ seeking health care yet being suspected of abuse had taken place during the years 2013 to 2017 and the selection criteria were: 1) all parents had been seeking health care for their infants; 2) all infants had been investigated according to a SBS/AHT-protocol with either findings of subdural haemorrhage, or radiologic findings indicative of abuse (fractures of rib, long bone or clavicle); 3) a paediatrician had mandated a report of suspected abuse to Social Services; 4) all infants had been subjected to measures according to the Social Service Act or the Compulsory Act (all but one had had Out-of-home care for a minimum of 10 days to up to 5 years, and one infant had been with his parents and siblings in a Social Services Investigative Custody Home); and 5) all parents had been interrogated by the police. In addition to meeting these criteria, almost all parents (10) had been arrested; one father was convicted of serious infant abuse in the District Court but found not guilty in the Court of Appeal; all charges against the parents according to the Social Service Act or Compulsory Act had finally been dropped; and all parents had, at the time of their interview, regained custody of their infants from Social Services, or were in the process of regaining custody of their infants.

The research team members had various pre-understandings of the studied phenomenon. Two team members have conducted medical epidemiological research within the field and have been court witnessesgiven court testimonies in related cases (UH and GH). One team member had experiences of providing peer support and advocacy group work related to this theme (GE). One team member had no previous research experience of this theme (ÅW).

### Data collection

The informants were contacted by telephone, and were informed about the study aim and the interview process. The data collection took place from April 2017 to January 2019. Informed oral and written consent was obtained. The parents were interviewed by one of the authors (GE), an experienced clinical psychologist and psychotherapist. An interview guide was used, covering areas relating to their reason for seeking health care, the consultation, the professionals’ suspicion of abuse, the events that then followed, the parents’ reactions and coping strategies, and their present situation (S1 Appendix). Both the mother and the father were interviewed at the same time. All interviews were conducted in their homes. A gentle and compassionate interviewing technique was applied [29, 30]. The topics from the interview guide were covered narratively by the informants once the interview had started with minimal prompting from the interviewer. The interviews lasted from between 3 and 4 hours and were audiotaped.

### Data analysis

The interviews were transcribed verbatim. The transcripts were imported into the qualitative text software OpenCode (https://www.umu.se/institutionen-for-epidemiologi-och-global-halsa/forskning/open-code/).

Qualitative content analysis was used for interpreting the data. This is an inductive approach characterized by searching for patterns, similarities and differences in the data [24, 25]. During the analysis of the interviews, meaning units were identified, describing a particular content or context. The meaning units were coded. The codes where then clustered for the development of categories and subcategories, illustrating the manifest meaning of the text. A theme was then created with the aim of understanding the underlying meaning. This synthesis of the interpretation was developed through close collaboration within the research team members. Data analysis continued until we had reached saturation, in which the described patterns were recurring, and after the final interview, which clearly confirmed our results. Hence, we determined there was no further need to recruit more interviewees. Our results were also presented to the participants for comments.

### Ethical considerations

The participants were informed that their participation in the interviews was voluntary and that they could end the interview at any time. They were informed that all material would be protected and handled confidentially and that only researchers in the project team would have access to the information and that no results would be presented in which any one individual could be identified. The interviewer was aware of the sensitive nature of the topic. Efforts were made to create a compassionate and empathetic atmosphere during the interviews. Precautions were taken because of the presumed potential vulnerability of the interviewees. and potential need of support. The study was approved by the Regional Ethical Committee in Uppsala (2017/099).

## Results

Two families had only had their first child (1 & 2), and four families already had older children before the event (3, 4, 5 and 6). Three of the parents were aged 25 to 29 years, and the others were aged 30 to 34 years at the time of the event. Three mothers had completed 12 years in school, and three more than 12 years. One father had completed nine years in school, one 12 years, and four had more than 12 years. Five of the infants were single born, and two were twins. Four were born pre-term. Infants’ ages at time of diagnoses of abuse were between 3 and 5 months. The families lived in six different counties in the catchment area of two different child protection teams within university hospitals.

Based on the analysis, an overarching theme, ‘Fighting for the protection of their child after being trapped by doctors’, was developed to represent the underlying meaning of the experiences, reactions and interpretations of the parents’ trajectories when being accused of shaking their infants. The first subtheme, ‘Being accused of injuring the child’, reflects how they sought care for their infants but instead, their own care of their infant ended and they were accused of maltreatment. The second subtheme, ‘Chaos and powerlessness’, refers to the parents’ emotions when losing custody of the child and being caught in the enforcement of legislation by the authorities. The third subtheme, ‘The unified fight against the doctors’ verdict, illustrates their fight to prove their innocence, their worry for the lost child, and the support they received to resist. The fourth subtheme, ‘The wounded posttraumatic growth’, considers the emotions and reflections in the aftermath, such as grief, lasting mistrust and panic, challenges to reunite the family, and the authorities’ inability to reconsider, but also their growth, both personally and as a family. The results are also illustrated in Table 1.

**Table 1:** Overarching theme: Fighting for protection of their child after being trapped by doctors, with sub-themes and categories.

Sub-themes and categories	Overarching theme
**Being accused of injuring the child** • Seeking care for their child • Not understanding the reason for extended investigations • Despair in not being listened to • A closing door and the end of care • Struck by lightning in being transformed into a suspected criminal	**Fighting for the protection of their child after being trapped by doctors**
**Chaos and powerlessness** • Being caught in the enforcement of legislation by the authorities • The shocking false “truth” in setting up the father • Severance from their infant opening an abyss • Struggling against emotional breakdown • Everything is turned against them • Censoring the infants’ records	
**The unified fight against the doctors’ verdict** • Desperate but controlled anger • Persistent worry for the child out of reach • Mobilisation through peer support and advocacy groups • Amassing second opinions to counter the accusations and the sudden disappearance of hope-eliciting professionals	
**The wounded posttraumatic growth** • Grieving for the lost time with the child and damaged attachment • The ambiguous insight that the child is also attached to the foster parents • Social Services not supporting family reunion • Authorities’ inability to reconsider, with apology or support from healthcare or social services • More courageous, compassionate and outspoken • The stigma, harrowing panic and enduring loss of trust	

### Being accused of injuring the child

The first sub-theme, ‘Being accused of injuring the child’, illuminated the shock when the parents sought care and instead were accused of being a perpetrator. The stories they told were about parents seeking help for emergency care relating to the condition of their young infants, or because they were worried about certain symptoms. They all described having had confidence with healthcare services. Gradually, or suddenly, they experienced a shift in their perception of the healthcare encounter; from a trustful consultation, to perceiving an agenda of distance, like the closing of a door. Their child was taken away for investigations they did not understand, and for which they were not provided an explanation. A creeping concern for what was going on, then not being listened to, which then evolved into panic, like being struck by lightning, occurred when they were then accused of having physically abused their child, having their child taken away, and being arrested.

Reasons for *seeking care for their child* included: concerns about skin lesions/rashes/bruises (3), being smaller in size, and having a lower weight gain than older siblings (1), vomiting and convulsions (1), agitation and crying, and other symptoms of pain from the baby (2). Some parents sought care several times, or explicitly asked for referral because of persistent worry that something was seriously wrong. The first level of care approached included the child care centre (2), primary care, and emergency care at a district, county or university hospital (4). In all cases, child protection doctors were consulted, either locally or at a university hospital.

Initially, the families were well attended and their infants were examined appropriately. In some cases, the parents were reassured and told not to worry. At a certain stage the scene changed, for some, instantly on their arrival at the referral hospital, where the diagnosis of suspected abuse had already been determined, and for others, this occurred more gradually. A common experience was *not understanding the reason for extended investigations* that continued, such as repeated radiology, ophthalmologic examinations and blood sampling. One couple expressed relief but then these extended investigations also increased their worry of their infant’s health; “Could it be a fatal disease?” (Mother_3_). None of those parents who were interviewed had been asked to provide consent for neuroimaging. Some parents were present during the neuroimaging, and experienced that the sedation provided to the infant was insufficient and that the infant eventually needed to be administered anaesthesia. Some were left alone while the child was taken for an x-ray and were never informed that neuroimaging had been done. None were informed that the siblings were also taken for extended x-rays or neuroimaging. The parents did not understand the reason for these procedures, nor did the doctors answer any of their questions. The infant was taken abruptly for the next investigation. “And why? It felt like we were not allowed to know the reasons … In hindsight we realized they were just following a standard protocol.” (Mother_1_).

Soon the parents felt *despair at not being listened to*. They were not asked for their explanations or their interpretations of the findings obtained by a radiologist. “It is quite horrifying actually, it can be from x-ray images without having talked with the parents, to make a diagnosis without having met the patient” (Mother_2_). Repeatedly, families asked for extended investigation for diseases that later were actually proven to be the cause of the findings. “We were ignored when we suggested specific tests should be carried out on her, ‘No, but we have no time now’” (Father_2_).

In an instant, almost immediately at referral or after prolonged investigations, the healthcare professionals decided on a diagnosis of abuse. This decision-making process was perceived as *a closing door and the end of care*. In several cases, the parents perceived that a child protection doctor led the investigations from a distance. The parents felt that nurses changed their attitude from being caring to being hostile, and some even made inappropriate innuendos. Once abuse was determined to be the sole cause, the momentum stopped and the doctors claimed that all possible investigations had been completed, while the parents desperately appealed to the doctors to make further investigations.

“I was trying to get the doctor to understand that I do not have mistreated children and tried to make them do a proper medical investigation […] that they needed to leave no stone unturned […] I was waiting all the time for when they had examined clearly and would discover that this is not abuse, coming to the conclusion that it is something with the skeleton that is not normal. They were so small and thin.” (Mother_5_)

The definitive abuse accusation was perceived as being *struck by lightning in being transformed into a suspected criminal*. The decision was communicated to the parents formally, harshly and without explanation, and, in several cases, in the absence of a responsible doctor. In some cases, the findings of the investigations were withheld from the parents by the doctors, and these were only communicated to them later by social workers.

The parents experienced the decision to report them to Social Services as being surreal. “You can't really understand, it must have been the wrong paper, therefore, wrong children […] They must have confused the x-ray with someone else. This is not true!" (Mother_4_ & Father_4_)

After the doctors made their report, Social Services acted promptly to take the infants, and their siblings, into custody. The parents were called into a meeting, then asked to follow the doctors into a separate room where police and social workers suddenly entered. “Then two additional police officers entered and someone from Social Services; ‘Yes, you are arrested.’ I don't understand anything, I'm just sitting with my child in my arms and breaking down. […] the woman from Social Services says, ‘We are going to take your child into custody’” (Mother_1_).

### Chaos and powerlessness

The second subtheme refers to the emotions that parents experience when they lose custody of their child. The experiences of being accused when the parents were innocent were about oppression, loss of integrity and powerlessness. The loss of the child evoked emotions of extreme longing, fear, desperation and hopelessness. Emotional breakdown was kept away because of an inner strength that came from fighting for their child.

*Being caught in the enforcement of legislation by the authorities*, such as police, prosecutors and Social Services was described as being tossed around in a tumble dryer. Being placed under arrest was a horrifying and degrading experience. “At one point I had a panic attack so I sat as well just shaking and rocking back and forth, when one of those guards came in and poked at me with his foot, saying only ‘It doesn't get better if you go off, shut up now,’ and then went out” (Mother_4_).

The parents were separated and isolated with no contact with the outside world. The first interrogations were performed without the presence of legal representatives, and were harsh and brutal. They were told “We know that you are 100% guilty” and were subjected to intense pressure and questioning. “It wasn’t until after the police interrogation that I fully realized that we could be convicted in spite of our innocence. I was completely shocked when I realized how the system works” (Mother_6_).

*The shocking false “truth” in setting up the father* was a common experience, as very early in the police interrogations the father was targeted as the perpetrator. “You’d better confess now. All other possibilities but SBS are outruled” (Father_5_). The parents perceived that the police tried to antagonize them, fishing for evidence of partner abuse, and trying to persuade the mother to apply for custody.

The loss of the child evoked emotions of fear, panic, despair and hopelessness, with the *severance from their infant opening an abyss*. “I felt like something had died in me, to be stripped of my own flesh and blood, it is not possible to describe the feeling you get, when we tried to have children for so long and then, they just take the child away from you” (Mother_2_).

Experiences of *struggling against emotion*al *breakdown* were described. However, the parents also described an emerging inner strength. “This feeling of … overwhelming hopelessness … can't breathe” (Father_4_). “It was very difficult, I tried to stay strong. It was not an option to break down” (Mother_5_).

The overwhelming experience was that *everything is turned against them*. The parents perceived that the investigations, interrogations and statements were being interpreted to fit the doctors’ verdict. Unanimously, the parents experienced being denigrated by Social Services. If fathers or mothers did not show their emotions, this was turned against them. “You are in shock, not your usual self, but the doctors, nurses and social workers are sure that we are abusers and have their judging glasses on […] They described me page-up and page-down, as mentally disturbed. That I don't have any attachment to my children and being emotionless” (Mother_5_).

The parents’ fight for their infants started after they had been released from custody. Soon they realized that the healthcare professionals were *censoring the infant’s record*. “We demanded several times to have access to our child’s record, but were denied this as it was stated to be part of a secret investigation protocol” (Mother_4_). In several cases the lawyer had to request a copy of the child’s record by multiple court orders, section by section.

### The unified fight against the doctors’ verdict

The third subtheme illustrates the parents’ fight for their innocence, their worry for the lost child, and their struggle to get their child back. The emotions of despair and worry for the child were transformed into controlled anger. This process was aided by social mobilization, attorneys and second opinions.

The false allegations evoked resistance. This emerged as a *desperate controlled anger*. The parents’ experience of being innocent but accused of child abuse and understanding that you are guilty until proven otherwise provided the momentum to resist and defend their family. “My thought as a mom was never give up my child … We are right” (Mother_2_). The parents described having adopted a double-sided strategy; 1) one external world in the fight to keep up the façade, avoid showing anger, and avoid saying too much, and 2) one inner world of sorrow and despair. “Never show any aggression. Show heart, intelligence. If you show the slightest aggression, you lose” (Father_1_). The family who had to agree to be detained at the Social Services Investigative Custody Home for monitoring with their three children for 10 weeks described their experiences: “It was a bit like in the film, *Life is Beautiful*, with this father who is in a Nazi concentration camp with his son trying to create the idea that this is just for fun” (Mother_3_ & Father_3_).

The parents had a *persistent worry for the child out-of-reach*. The pain of being denied their ability to protect their child was almost unbearable. “We felt so sorry for her, we thought of her constantly” (Mother_2_ & Father_2_). One breastfeeding mother described how she was offered medication to stop breastfeeding when she was arrested. “Then I was mad and reacted in a way, saying ´Knock it off, we’ll get out of here and my child will be breast fed’” (Mother_1_).

The parents’ extended family became united and worked hard in the fight; fathers and mothers, grandmothers and grandfathers, stepmothers and stepfathers, and siblings. This was experienced as *mobilization through peer support and advocacy groups*. All of the family members were full of anger and assisted by googling and gathering information and being a source of support at all visits with the authorities. Some moved into the homes of the accused to be nearby. The extended social network of the affected families was extremely important; friends, old colleagues, relatives who were social workers or police officers, past teachers, lawyers in their social network, and members of their church congregations. “My sisters are googling the whole night, and … checks the cell phone and the computer all the time. We're talking, I'm talking with several callers on the line. […] Mom and dad accompanied us to all the meetings” (Mother_3_).

All of the participants described having gained support from the advocacy groups, including emotional and second opinion support, guidance on how to proceed, and the importance of continuously documenting the history and record of all encounters with Social Services. *“*If we hadn't worked on this very hard and hadn’t had such support and the entire network and find this help through the RFFR. I mean where would you end up? In jail and the children would be in custody. Our daughter might come home then … but the twins would be gone, it is a fact” (Mother_5_). Many of the parents’ intuitive perceptions of the underlying medical conditions that contributed to the findings at the very start of the diagnostic process were confirmed by *amassing second opinions* and, as a result, the parents were exonerated in court.

One mother described an unpleasant and disturbing experience of the *sudden disappearance of hope-eliciting professionals*. One orthopedist commented on the newborn child’s diagnosis of rickets and the older child’s diagnosis as abused; ‘“But this is not correct. It must have been a wrong diagnosis with the previous child,’ [she said] … And she talked with us and we met with the child and she checked up a lot, and she was really on our side. Until one day when she had talked with child protection doctor in the university hospital, and all of a sudden she could not do anything because it was child abuse … so she turned into a really completely different person” (Mother_4_).

Several participants experienced that other doctors questioned the child protection doctor’s views, but none of them came to challenge the medical hierarchy, as one participant explained. “They are only servants, obey to the orders” (Father_1_). Only one professional, a child care nurse, supported the parents in court. Some participants experienced attitudes of other professionals who understood their innocence, such as the police investigator, and prison officers, who suggested, “This is wrong, you shouldn’t be here” (Mother_3_ & Father_3_). Some participants experienced that social workers assured them, off the record, that they trusted them as parents and that they would push to get the children back promptly, but nothing happened.

### The wounded posttraumatic growth

The fourth subtheme describes prevailing symptoms of posttraumatic stress symptoms, such as flashbacks, panic and anxiety. Grief for the lost time with the child and challenges to reunite the family were very much present, as was a lack of support from Social Services in this regard. Personal growth by having to be more out-spoken and courageous in respect of their understanding of the outer world was described. Unanimously, the parents had experienced authorities’ inability to reconsider and expressed a deep mistrust of paediatric care.

The participants, having had their children in Out-of-home care for months, or even for years, suffered in *grieving for the lost time with the child and damaged attachment*. “So much happens in such a short period of time when they are small. That time is lost for us. We missed all of it. So we may get to know a completely new, thus, we have to get to know our son as a completely new boy!” (Mother_6_). One mother feared that the forced separation would become embodied and would hurt the child later on. Parents who had several children and who had been separated from them for years experienced damaged attachment with difficulties in the family reunion. Parents reported feeling alienated from the child who re-entered the family; who is this, and how do I get to know the child? This feeling also influenced the parents’ attachment to the returning child by bringing out emotions of uncertainty, being overprotective and fear of making mistakes. “… the feeling is wrong, she will be somewhere in between […] It hurts so much. You are so afraid for it to be wrong […] She has missed her older siblings, they've missed her growing up … When I spend time with my children today, and I see that the other children directly in a second just take what they want and do what they feel. But it doesn't work for her. For even if she is mine, she is not mine” (Mother_4_).

One consequence of the family reunion was *the ambiguous* insight that the child is also *attached to the foster parents*. “He cries because he does not want to come here, and he cries when he leaves us” (Mother_6_).

The participants reported in most cases that *Social Services did not support the family reunion* after the judiciary decision, and that they delayed, and even opposed, the transition from having the most restrictive contacts during Out-of-home care to the family re-union. However, some families experienced that the foster families were very supportive in this transition, while others experienced mistrust, disappointment and denigration. Several parents expressed that they perceived that Social Services had a hidden agenda with the foster parents and had presumed that the Out-of-home care would be permanent. “We understood later that that Social Services had promised the foster family that our child would be placed there permanently” (Mother_2_ & Father_2_).

Because the parents had been exonerated, they experienced the *authorities’ inability to reconsider*, and received *no apology* and no *support from healthcare or Social Services*. An overwhelming experience described by the participants was that their families had been injured, that they had been through a terrible and horrifying trauma; an act of abuse by healthcare services. No doctors, no hospitals, none of those who had accused the parents of being abusers came back to them afterwards to support them. “Demolishing a wall, absolutely anyone can do. But they will not help to build it up again. When they have been wrong, or they don't want to admit they have been wrong. It becomes the prestige instead […] instead of, ‘We thought we didn’t do correctly,’ or ‘We didn’t know any better, but we have learned now’” (Father_4_).

Some social workers admitted their wrongdoings off the record, but Social Services offered neither an excuse nor support, the parents were simply told, “We have done nothing wrong”. Several participants expressed that they would have wished to have had supportive meetings with the authorities. Two of the families had registered complaints with the Health and Social Care Inspectorate (IVO), but only received the response that no wrongdoing had been identified. One father had fantasies of revenge, such as going public with the identity of the doctors who falsely accused them of abuse to warn other families. One family wanted to sue the perpetrating doctor through the courts.

Many of the participants, both fathers and mothers, described how the experience had changed them positively; to be *more courageous*, *compassionate and outspoken*, having increased confidence, being more reflective and questioning more, getting more upset about injustices, being more empathetic, and less biased in their attitudes. “My mom said a very good thing to me a few months ago, she said, ‘When you were little you were always curious, you were fearless and whatever you wanted to find out or do, you went for it. Then something happened during your years in school … but now I recognize that spirit again.’ I'm less anxious, more brave and curious. I have no inhibitions in that sense now” (Father_1_).

The *stigma* of their experiences continued to pursue the participants. Some had changed workplace and domicile, to protect themselves and the child when they were grown up. A *harrowing panic* continued to haunt them, both in daily life and in nightmares. Several had endured depressive and anxiety symptoms, re-living the trauma repeatedly, for which they sought therapeutic support. Extreme focus was needed in order to manage the process, otherwise, “all the held back” emotions were opened up. “So I have had some setbacks … Everything catches up with you in the end. Yes, I tend to blend in and focus, but my feelings always catch up with me” (Father_1_). Emotions of panic hounded many of the participants. Just passing by the police station, the Social Services office or the hospital could evoke flashbacks of the trauma. Two families dismantled their doorbell afterwards in fear of intrusion. “I can still wake up at night, having flash-backs, wake up completely horror-struck … as soon as I see the Police station or the Social Services office, the panic is close […] When I hear motors running outside and I think they are coming to detain us” (Mother_1_).

*The enduring loss of trust* was very prevalent in the interviews. Unanimously a fear, for some, a phobia, of seeking health care for their children was expressed. “I still have a phobia of visiting doctors and hospitals with the children, I do not want it, you dare not go in with children. It falls over on you because I can’t do it, it is not possible. Well, I went to the allergologist with them, but then my stepfather accompanied me (Father_5_).

Several of the mothers expressed such a profound *loss of trust* in healthcare services that they were terrified of having another child because of what they had been through and the fear that the authorities would take that child as well. One mother had even had an abortion because of this. “I carry a sadness that I, because I long so much for a small baby, but I dare not … I never want to go through this again” (Mother_6_).

One mother made a decision to have another child despite reluctance from her husband and a huge fear from her mother-in-law that the newborn would also be taken into custody. The new child was born preterm, as was the first. The mother refused to leave the hospital until a proper investigation was completed, which the doctors finally did, and then came rushing in as severe osteopenia was revealed. As a result of her persistence, this mother regained custody of her older child who had erroneously been diagnosed as being abused when, in fact, the infant had suffered from bone fragility.

## Discussion

### Key results

The parents’ experiences of seeking health care for their infants and instead being accused of abuse are reflected in their narratives of shock, losing their infant and being caught in the hostility of the authorities. The descriptions of their experiences included a fight for survival against emotional breakdown and family disintegration by mobilizing inner forces, social support and second opinions to finally be exonerated from the allegations and regaining custody of their infants. The aftermath of these events, without any apology or support from the authorities, included feelings of sorrow and bereavement for the lost time with their child, challenges with family reunion, and long lasting post-traumatic emotions, such as anxiety, panic and distrust of the authorities, but also a sense of personal development in being more courageous and outspoken.

### Strengths and limitations

Trustworthiness, the concept that the participants’ voices are heard, is the essence of qualitative content analysis [24, 25]. It is possible that the interwier's pre-understanding of the subject is a bias. However, the study guide was developed in collaboration with a sociologist outside the research team. Furthermore, the interviewer's (GE) pre-understanding could also be considered a strength and possibly even a necessity, as well as her skills as a clinical psychologist. It is unlikely that all parents would have participated without complete trust in the interviewer, something that was likely linked to her pre-understanding. Hence, the topics from the interview guide were covered narratively by the informants with minimal prompting from the interviewer. Finally, the main contributor (ÅW) during the analysis has no previous experience in this subject but have expertise in analysis of in-depth interviews with health professionals being traumatized in work. Thus, we consider it is unlikely that interview bias has influenced the interpretation of the interviews. Credibility and dependability were pursued through a reflective dialogue in the research team during the analytic process. Participants’ recognition of the finding supports confirmability. Regarding transferability, one bias can be that our participants had an advantage in respect of their educational background and civil status in contrast to families in general who have infants meeting any of the criteria of SBS/AHT concomitant with entry into Out-of-home care during the years 1997–2015 in Sweden (n = 182) [19]. Further, our participants had not experienced divorce and all had regained custody of their infants. One bias could also be the small sample size. We cannot fully know the reasons for the couples (4 mothers and 4 fathers) that declined to participate, but we can make some inferences based on the experiences of those who did participate. When the interviewer (GE) made contact, she was only able to reach mothers of those couples that declined. The mothers told her that they and their partners still experienced a total lack of trust in “unknown others”, particularly those where the fathers had been sentenced to prison (2 couples). They all expressed that they had tried very hard to repair their relationships and their family and move on. Thus, we do not consider that the non-participants’ experiences would have been significantly different from those being interviewed, at least in their not having had any less traumatic experiences.

Nevertheless, we consider that our results might still have bearing on other parents who have experienced being falsely accused of abuse, and also for parents in general who are trying to care their best for their children.

### Interpretation

Four of seven infants were pre-term born. Prematurity is considered as a risk factor for maltreatment[31]. However the criteria of SBS/AHT, as subdural haemorrhage and rib fractures and long bone fractures, are more prone among pre-term borns [15, 16] and circular reasoning can bias their association to SBS/AHT diagnosis. Instead, it might be the case of more health care seeking among parents with pre-term because of infants`needs, and anxiety and stress in the caring [32].

The trajectories described by our participants reveal the potential hazards of a child protection system that is characterized by proceduralization and technicalization in a climate of fear, mistrust and blame [6] that leaves little room for clinical thinking and reconsideration. The techno-science for diagnosing infant abuse such as SBS/AHT, purportedly for the sake of the infant, has inherent fallacies [8]. This is even more pronounced as the triad of SBS/AHT is shown to have insufficient evidence to be caused by shaking [13], although these findings have been challenged by vivid criticism its conclusions has not been changed and are considered to be valid [14, 33–36].

Our participants, when seeking care for their infants, instead experienced harm inflicted by healthcare services. The diagnostic error had disastrous consequences for the children and their parents. The parental experiences expressed in this study are in accordance with patients’ narratives in cases of diagnostic errors; ignorance of patients’ knowledge; and disrespect, manipulation and deception [37]. Further, our informants were never informed, nor did they give their consent, about the extended skeletal x-rays and computed tomography that had been performed, which is contrary to Swedish Law (Patient Act 2014:821 & Health Care Act 2017:30). Promotion of patient safety is paramount in the prevention of harm in health care, and when an adverse event takes place there is a professional duty of candour [38]. A desire for an apology, support and appropriate inquiry is expressed by patients who have been exposed to medical errors [39]. None of the participants here experienced any of these, which might have added to the protracted suffering afterwards, a finding that is consistent with the experiences of parents in the USA [17]. Threats of being sued in court by parents have been reported by a doctor who had reported suspicion of abuse [4].

“The Monstrous Perpetrator” is an underlying discriminatory stereotype often enmeshed by gender, race and class, and is often described in connection with SBS/AHT [8, 17]. This could be understood in the urgency to act when the pediatrician is trying to make sense of a baby who has x-ray findings suggested to be caused by shaking according to the SBS/AHT-paradigm. The Swedish context, with its relatively high percentage of fathers having parental leave, might have contributed to the finding that the experiences and emotions of the mothers and fathers were shared in general, although those of the fathers were more targeted by the judiciary process, while in the US context the blame is focussed more on the mothers or nannies [17].

Loss was the overarching theme presented by Zeman (2004); the pain of the “physical separation”, “the loss of fantasy that they could protect their child”, and “the loss of the sense that their parenting experience had been normal” [23]. These findings align with our categories; *Severance from their infant opening an abyss* and *Persistent worry for the child out-of-reach* and *Grieving for the lost time with the child and damaged attachment*.

The “medical fact of abuse” presented by the doctors to the judiciary and Social Services reversed the burden of proof and placed an exceedingly severe strain on the participants. Such experiences, contrary to a normal court process, are shared by Hansen et al., who reported that “Families are guilty until proven innocent” [21]. Further, the finding that Social Services are overly biased to justify the need to place children in Out-of-home care according to “medical fact” is also confirmed by Hansen et al., who suggest that evidence presented by child protection caseworkers “is often only partially correct and may include rumour, gossip and innuendo about parents” [21]. In the US, the consequences of judicial authorities’ judgement on the basis of a medical diagnosis of SBS/AHT have received much attention in respect of flawed convictions [17, 40].

Noteworthy, several of our participants met professionals as doctors, police or social workers, who expressed to the interviewees that they perceived that this process could be erroneous. However, none of the participants experienced that those professionals were then willing to act to help the families and instead realigned themselves under the direction of an authoritative system. This illustrates a potential danger of a system working strictly according to guidelines under a leadership of often distant experts with little or no face-to-face meetings with the accused parents [41], not facilitating opportunities for reconsideration during diagnostic procedures [42]. In a decentralized health system, one that is family centred, clinicians may act with more independence after having experienced that “What a family goes through with a CPS investigation, when the case is unfounded, is traumatic” [2].

Mistrust in seeking health care is often described after a medical error [43]. In the study by Zeman (2004), falsely accused parents’ “loss of fantasy of their faith in the system” was found to be another of the four losses [23]. Our participants felt betrayed by the system, describing it as being like an earthquake in a reality that they had trusted. Mistrust in healthcare services was unanimously explicit in this study and persisted as a long-lasting emotion, with the participants even resorting to abortion or avoiding getting pregnant to elude further trauma.

Our findings do show that the traumatic event of child custody loss did have long-lasting impacts on the parents’ lives, as previously shown [22]. The emotional trauma that the participants experienced after being exposed to a traumatic event went “generally beyond the realm of normal human experience” and were examples of “a stressor that would evoke significant symptoms of distress in almost everyone” [44]. Several of the informants were close to emotional breakdown, but survived during the battle for the protection of their child and the family. However, they still suffered with PTSD-symptoms afterwards, such as panic and flash backs. Resilience to the trauma exposure was facilitated by the strong social support that the participants received and may also have contributed to the personal growth that several described [45].

As this was a qualitative study, the longer-term consequences for the parents cannot be measured. A study by Wall-Wieler et al. (2018) indicates an increased risk of suicide attempts and completion among mothers after a child custody loss [46]. The families in this study were severely traumatized, although they were reunited. A study analysing the long-lasting psychological suffering of Swedish parents who have been exposed to Out-of-home care, whether being accused of SBS/AHT or not, is underway.

Our results, illustrating a system failure within child protection, have important clinical implications for the practice of reporting suspected abuse and avoiding false allegations. A techno-science that is dependent on a diagnostic procedure that lacks scientific support cannot be enrolled as the grounds for a fast decision to justify Out-home-care. Adequate training [1–3, 5] must be evidence based according to up-to-date knowledge. Increased reasoning is required among doctors and nurses to determine how much more is needed from mere suspicion to establish that a child has been abused. Social workers and psychologists should be involved at an early stage. The Swedish Social Service has implemented *Signs of Safety* strategies when a report of suspected child abuse is received, to consider whether placements with relatives could be an alternative during the investigation [47]. None of our participants experienced that alternative. Except in life-threatening emergencies, it must be time to consider the justification before taking a child from its home, as the first obligation is to do no harm. This position is expressed by the World Health Organization and International Society for Prevention of Child Abuse: “The least detrimental course of action for the child, and the least intrusive one for the family, should be employed, as long as the child’s safety is assured […] The removal of the child from the home and placement in a relative`s home, a foster home or–as a last resort only–residential care.”[48]. Further, the collateral damage inflicted when a child is “adopted” by another family on false premises warrants follow-up support from Social Services to the four parents in coping with the trauma and in the best interests of the child during the child’s upbringing.

## Conclusion

To conclude, being innocent but accused of SBS/AHT evoked emotions of intense stress but the parents survived because of a successful fight to regain custody of their child. However, the trauma had a long-term impact on their lives, as they still suffer from posttraumatic stress symptoms and a long-lasting mistrust towards healthcare services and other authorities. The results provide important inferences for the need to restore system failures in child protection services.

## Supporting information

S1 Appendix Interview guide. (DOCX)
