# Supplementary material for: Parents’ experiences of seeking health care and encountering allegations of shaken baby syndrome: A qualitative study
Source: PLoS One. 2020 Feb 20;15(2):e0228911. doi: 10.1371/journal.pone.0228911 (PMC7032704; doi:10.1371/journal.pone.0228911)
Supplement: S1 Appendix — (DOCX) [file pone.0228911.s001.docx]

S1 Appendix. Interview guide

*Before the consultation*

1. Tell me what made you seek health care for your child?

*Visits to the doctor*

1. How was the consultation for you?

*Suspected*

1. Tell us how it was to be suspected?

*Events after the consultation*

1. Then what happened after the consultation and how was it for your family?

*Strategies*

1. How have you tried to handle the situation, and is there anything that made it easier for you?

*The current situation*

1. Tell us about your current situation.

*Closing questions*

1. Is there something new that has emerged in this conversation which you have not thought about before?
2. Is there something you want to emphasize as especially important for me to understand?
3. Do you have any further questions?
